# Ageing as a two-phase process: theoretical framework

**DOI:** 10.3389/fragi.2024.1378351

**Published:** 2024-04-08

**Authors:** Flaminia Zane, Claire MacMurray, Clémence Guillermain, Céline Cansell, Nicolas Todd, Michael Rera

**Affiliations:** ^1^ Université Paris Cité, INSERM UMR U1284, Paris, France; ^2^ Centre François Viète, UR 1161, Nantes Université, Nantes, France; ^3^ Université Paris-Saclay, AgroParisTech, INRAE, UMR PNCA, Palaiseau, France; ^4^ Eco-Anthropologie (EA), Muséum National d’Histoire Naturelle, CNRS, Université de Paris, Musée de l’Homme, Paris, France; ^5^ Université Paris Cité, Institut Jacques Monod, CNRS UMR 7592, Paris, France

**Keywords:** ageing, Smurfs, natural death prediction, modelling, two-phase ageing framework

## Abstract

Human ageing, along with the ageing of conventional model organisms, is depicted as a continuous and progressive decline of biological capabilities accompanied by an exponentially increasing mortality risk. However, not all organisms experience ageing identically and our understanding of the phenomenon is coloured by human-centric views. Ageing is multifaceted and influences a diverse range of species in varying ways. Some undergo swift declines post-reproduction, while others exhibit insubstantial changes throughout their existence. This vast array renders defining universally applicable “ageing attributes” a daunting task. It is nonetheless essential to recognize that not all ageing features are organism-specific. These common attributes have paved the way for identifying “hallmarks of ageing,” processes that are intertwined with age, amplified during accelerated ageing, and manipulations of which can potentially modulate or even reverse the ageing process. Yet, a glaring observation is that individuals within a single population age at varying rates. To address this, demographers have coined the term ‘frailty’. Concurrently, scientific advancements have ushered in the era of molecular clocks. These innovations enable a distinction between an individual’s chronological age (time since birth) and biological age (physiological status and mortality risk). In 2011, the “Smurf” phenotype was unveiled in *Drosophila*, delineating an age-linked escalation in intestinal permeability that presages imminent mortality. It not only acts as a predictor of natural death but identifies individuals exhibiting traits normally described as age-related. Subsequent studies have revealed the phenotype in organisms like nematodes, zebrafish, and mice, invariably acting as a death predictor. Collectively, these findings have steered our conception of ageing towards a framework where ageing is not linear and continuous but marked by two distinct, necessary phases, discernible *in vivo*, courtesy of the Smurf phenotype. This framework includes a mathematical enunciation of longevity trends based on three experimentally measurable parameters. It facilitates a fresh perspective on the evolution of ageing as a function. In this article, we aim to delineate and explore the foundational principles of this innovative framework, emphasising its potential to reshape our understanding of ageing, challenge its conventional definitions, and recalibrate our comprehension of its evolutionary trajectory.

## Graphical Abstract

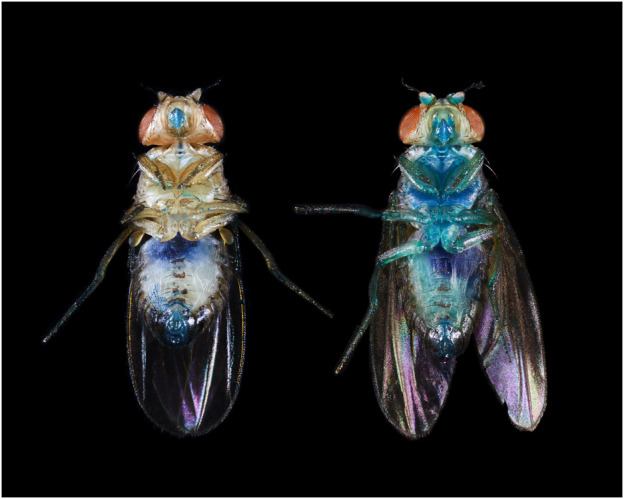

## Introduction

While without clear consensus (Cohen et al., 2020), ageing is generally defined as “a decline in function of the organism during adulthood” (López-Otín et al., 2023), “characterised by an [age-dependent] progressive loss of physiological integrity, leading to impaired function and increased vulnerability to death” (Lopez-Otin et al., 2013). This definition largely reflects the way we, as humans, experience ageing. However, there are limitations to its validity, especially as it implies that the process of ageing is only somewhat distinguishable from the mere passing of time (i.e., as time passes, an organism is ageing).

By placing time as central in ageing, the traditional understanding and modelling of this phenomenon does not address how the rate of ageing varies amongst individuals. Indeed, human-like ageing is most apparent demographically (when individuality/individual variance is not exactly captured well) and is seen as a force of mortality that steadily and predictably increases over a given lifespan, progressing exponentially with age (Gompertz, 1825). Consequently, this demographic perception of ageing strongly shapes our mechanistic understanding of the involved processes (i.e., the mechanisms of ageing are a phenomenon of chronology). Chronological age, while considered an “imperfect surrogate measure of the ageing process” (Horvath and Raj, 2018), is nevertheless still used as the main parameter of ageing, largely due to its simplicity. However, the need for non-invasive, conserved, quick-to-measure, and time-independent biomarkers of ageing has been recognized now for more than 30 years (Baker and Sprott, 1988; Warner, 2004). Such biomarkers are defined as “biological parameters of an organism that either alone or in some multivariate composite will, in the absence of disease, better predict functional capability at some late age than will chronological age” (Baker and Sprott, 1988). It is well observed, most notably in isogenic populations and highly controlled environments, that individuals do not age at the same rate (Zhang et al., 2016). This implies a difference between the chronological age of an organism - the time elapsed from birth—and its biological age—the age in terms of its physiology and associated instant risk of death.

Nine hallmarks of ageing—recently expanded to twelve—are broadly used by the ageing research community as molecular markers associated with biological ageing (Lopez-Otin et al., 2013; López-Otín et al., 2023). These twelve hallmarks were further divided into three categories: primary, antagonistic, and integrative. The primary hallmarks are processes affected by the accumulation of damage over time and, “unambiguously contribute to the ageing process”; this includes: genetic instability, telomere attrition, epigenetic alterations, loss of proteostasis, and disabled macroautophagy. Antagonistic hallmarks group damage-response processes that have beneficial effects when active at low levels, but damage the organism if continuously present (i.e., mitochondrial dysfunction, deregulated nutrient sensing, cellular senescence). Finally, the integrative hallmarks (stem cell exhaustion, altered intracellular communication, chronic inflammation, and dysbiosis) arise at a systemic level through the non-reversible combination of the primary and antagonistic markers. The hallmarks are evolutionarily conserved (Lemoine, 2021), alter with increasing age, and when intervened upon, the lifespan of an individual increases (Lopez-Otin et al., 2013). In 2018, a similar review of the literature was used to define six transcriptional hallmarks of ageing (Frenk and Houseley, 2018). While underlining the difficulty in defining a “consensus signature” across species and tissues, the six hallmarks defined here are: downregulation of mitochondrial proteins, downregulation of ribosomes, reduction in growth factor signalling, dysregulation of gene expression, dysregulation of immune genes and stress and DNA damage (for the last, they specify that data does not give way to a clear consensus, as in some cases the expression of DNA damage genes is above average, and in others, it is below). Notably, it has been recently shown that at least some of these six hallmarks display asynchronous and non-linear progress across tissues and organs in mice (Schaum et al., 2020). This observation only further provokes a sense of doubt if we are to continue conceptualising ageing as linear and coupled to the chronological life of the organism. To account for inter-individual variability during ageing, the concept of frailty was introduced in the late 20th century as a previously unobserved individual modulator of the force of mortality (Vaupel et al., 1979). Frailty was first adopted by clinical settings and subsequently, experimental biology (Faya-Robles, 2018). In these contexts, the concept was initially used to identify multiple frailty phenotypes; these phenotypes later developed into frailty indexes. The indexes are based on sets of biological/physiological/behavioural parameters and these parameters allow for the prediction of the mortality risk of an individual independently of their chronological age (Fulop et al., 2010; de Vries et al., 2011; Dent et al., 2016). Research that involves model organisms now make use of these indexes as well (Whitehead et al., 2014; Baumann et al., 2018; Heinze-Milne et al., 2019). The latest technological/conceptual advancement in this field is “ageing clocks”—e.g., the mammal epigenetic clock that identifies 5-cytosine methylation of CpG sites (Bocklandt et al., 2011; Hannum et al., 2013; Horvath, 2013; Horvath and Raj, 2018) and transcriptomic clocks in *Caenorhabditis elegans* (Tarkhov et al., 2019; Meyer and Schumacher, 2021). These are yet another attempt to discriminate between biological and chronological age.

If ageing as we have presented it thus far is complex and difficult to define, understanding its evolutionary origins has revealed extremely complex problems for the past 150 years. Although it was first seen as an adaptive force of evolution immediately following the publication of Darwin’s work (Weismann, 1882), most of the 20th century has seen the development and adoption of theories that propose that ageing is a mere by-product of evolution (i.e., not adaptive). Ageing, as it has been theorised, exists through evolutionary time not because it is advantageous nor is it a process in itself but because the age-dependent selective pressure creates a “selection shadow” (Haldane, 1941). The “selection shadow,” in theory, allows for the accumulation of 1) deleterious mutations acting only late in life (Medawar, 1952) or 2) genes with early life benefits showing negative effects on fitness, again, only later (Williams, 1957).

In this Hypothesis and Theory article, we present and discuss a decade of work on the Smurf phenotype, initially described in *Drosophila melanogaster* as an age-related increase of intestinal permeability. This increase in intestinal permeability is made observable by feeding the flies a blue food dye that leaks only in individuals about to die of natural causes, hence the name “Smurf” (Rera et al., 2011). As our work progressed, we ascertained that the use of such a phenotype allows for the identification of individuals, within a population, who will soon die from natural causes. These same individuals show the physiological hallmarks traditionally associated with ageing (Rera et al., 2012). This prompted us to develop a theoretical framework whereby ageing is made of two consecutive and necessary phases. The transition between these two phases can be detected experimentally by the Smurf phenotype. This phenotype distinguishes a first phase (in which the risk of reaching the point of transition increases with time) from a second phase, where individuals show the long-described properties of ageing. Notably, differentiating between Smurf and non-Smurf individuals within an ageing population allows for the deconvolution of the “ageing transcriptome” into its chronological and biological components (Zane et al., 2023). Thus, our conception of ageing better accounts for biological disposition and heterogeneity of a population. Using this framework, we have developed and published approaches that seem to reconcile some of the empirical vs. theoretical discrepancies mentioned in the aforementioned paragraphs. Precisely, we have modelled longevity curves of these two consecutive phases (that, as we have observed, constitute a lifespan). An abrupt transition is distinguishable and the parameters of this model are experimentally quantifiable (Tricoire and Rera, 2015). Additionally, this theoretical framework allows for the conceptualisation of ageing within evolutionary theory as something that has been and is directly selected, rather than a mere by-product of other processes under selection (Roget, 2018; Méléard et al., 2019; Roget et al., 2024).

## Body

### The Smurf phenotype is an *in vivo* marker of frail individuals

**FIGURE 1:**
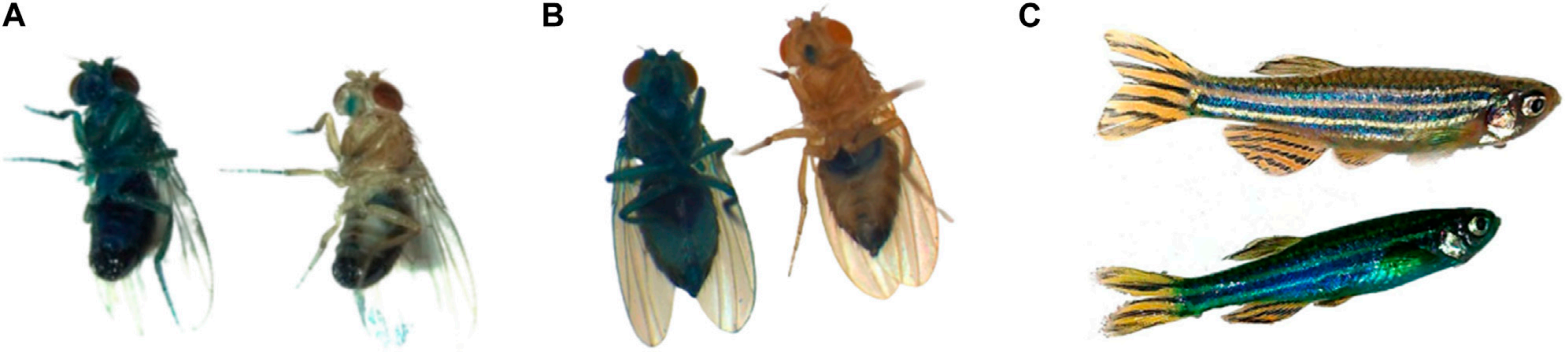
**(A)** Smurf and non-Smurf males, *D. melanogaster*; **(B)** Smurf and non-Smurf females, *D. melanogaster*; **(C)** Smurf and non-Smurf female, *D. rerio*.

We first described the Smurf phenotype in *Drosophila melanogaster* as an increased intestinal permeability to the FD&C blue dye #1, making the fly appear completely blue. It was observed, while assessing the food intake of flies fed the blue dye and frozen for subsequent quantification following the protocol from Wong et al. (2009), that these flies would turn completely blue upon thawing. We then observed that living flies also turned blue as they aged. This raised a number of interesting questions. It was finally determined that the Smurf phenotype occurs naturally, in all individual flies, as they age (Rera et al., 2011) (Figures 1A, B). The mechanism of the intestinal leakage is not fully understood, but published data by Resnik-Docampo et al. (2017) suggests that weakening of tricellular junctions somehow plays a role. Our recent transcriptome study (Zane et al., 2023) shows the deregulation of extracellular matrix related genes in Smurfs. Notably, we observed a downregulation of laminin (*LanB1* and *LanB2I*)—major components of the basal lamina whose mutations cause accelerated ageing in humans (Eriksson et al., 2003)—and collagen (*Col4a1* and *vkg*). Although we do not yet know whether the detected signal is specific to certain tissues—nor associated with a decrease of the corresponding proteins—as the data is from the whole body, it has been shown that *Col4a1 Drosophila* mutants present premature loss of intestinal integrity and increased inflammation markers in the gut (Kiss et al., 2016). Interestingly, the two ECM reshaping metalloproteinases *Mmp1* and *Mmp2* are also upregulated in Smurfs, indicating a possible remodelling of the extracellular matrix (ECM)—an emerging hallmark of ageing (Statzer et al., 2023), in this last phase of life. It is important to stress that the increased intestinal permeability is used here merely as a correlating biomarker rather than a cause of the biological characteristics we are describing.

The age-dependent increasing risk of becoming Smurf is conserved in other model organisms including other *Drosophila* species, the nematode *C. elegans*, *Danio rerio* (Figure 1C) (Dambroise et al., 2016) and the mouse strain AKR/J (Cansell et al., 2023), with a remaining life expectancy of Smurf individuals that is seemingly proportional to the organism’s life expectancy at birth. We indeed demonstrate that this phenotype is a harbinger of death across species, allowing for the identification of individuals about to die from natural conditions 2–3 days prior to death in *Drosophila* (Rera et al., 2012; Tricoire and Rera, 2015; Dambroise et al., 2016), 1–4 days in nematodes, and 6 months in zebrafish (Dambroise et al., 2016). More recently, we have shown that this phenotype helps predict impending death in the short-lived mouse strain AKR/J, approximately 2 weeks prior to death (Cansell et al., 2023).

Recent work from Livingston et al. (2020) has demonstrated that Smurfs not only show an increased intestinal permeability to the blue dye, but also a significant decrease in excretion through the Malpighian tubules, the fly’s kidney. This indicates that the Smurf phenotype encompasses changes in organ function beyond the gut. In fact, Smurf individuals show significant decreases of energy stores and spontaneous activity, high levels of inflammation markers including *drosocin*, *drosomycin* and *diptericin* (Rera et al., 2012) as well as strong decreases in fertility at the T_50_ (the half-life of the population)—75% in non-Smurfs versus 5% in Smurfs after adding young males (Rera et al., 2018). These observations suggest a systemic set of changes co-occurring in this last/Smurf phase of life.

**FIGURE 2:**
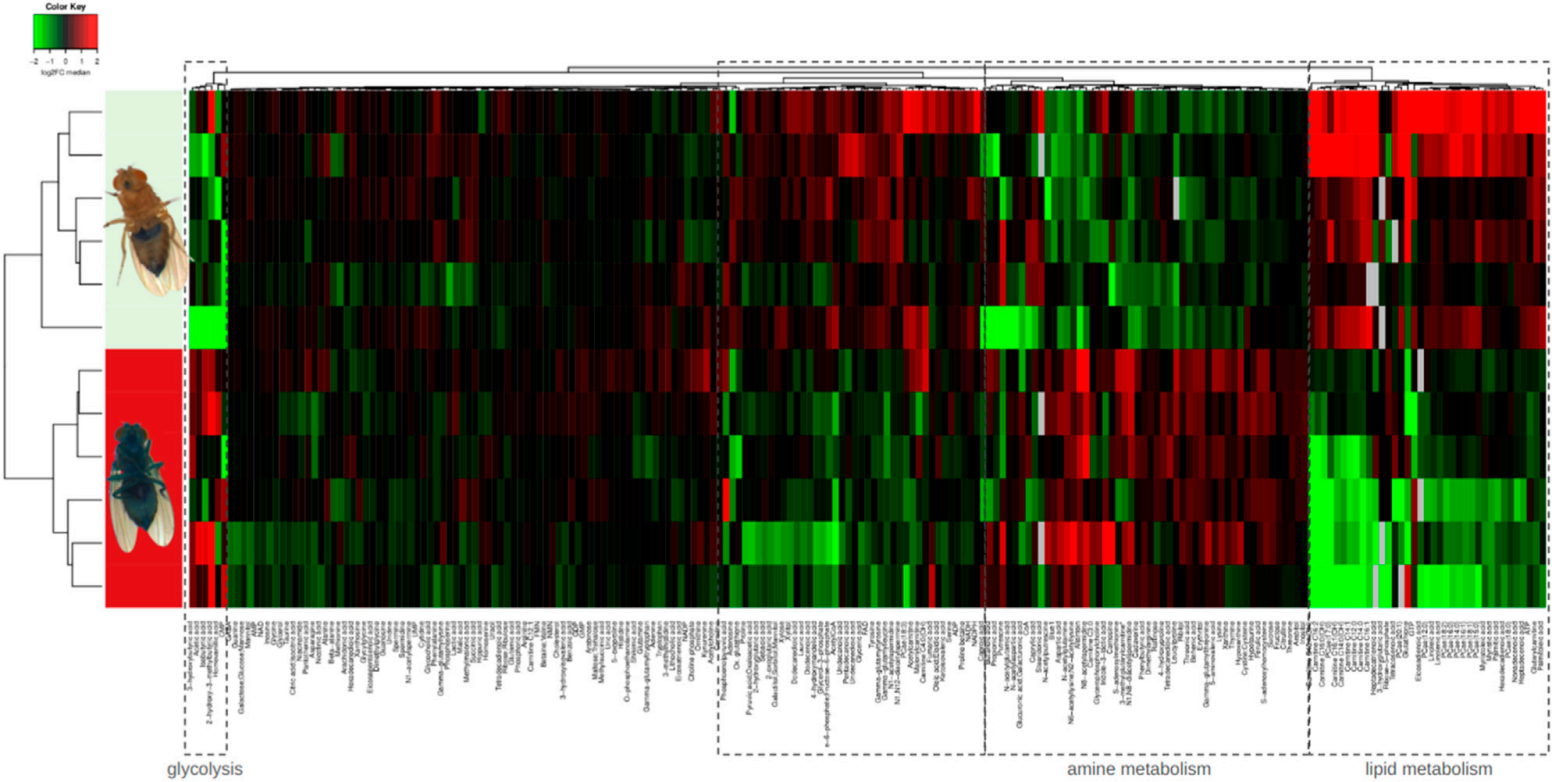
Unsupervised hierarchical clustering of whole mated DGRP26 female flies allows binary classification of Smurfs and non-Smurfs. Smurf females show an increase of glycolysis intermediates and end-products and significant decrease of lipid metabolism intermediates.

**FIGURE 3:**
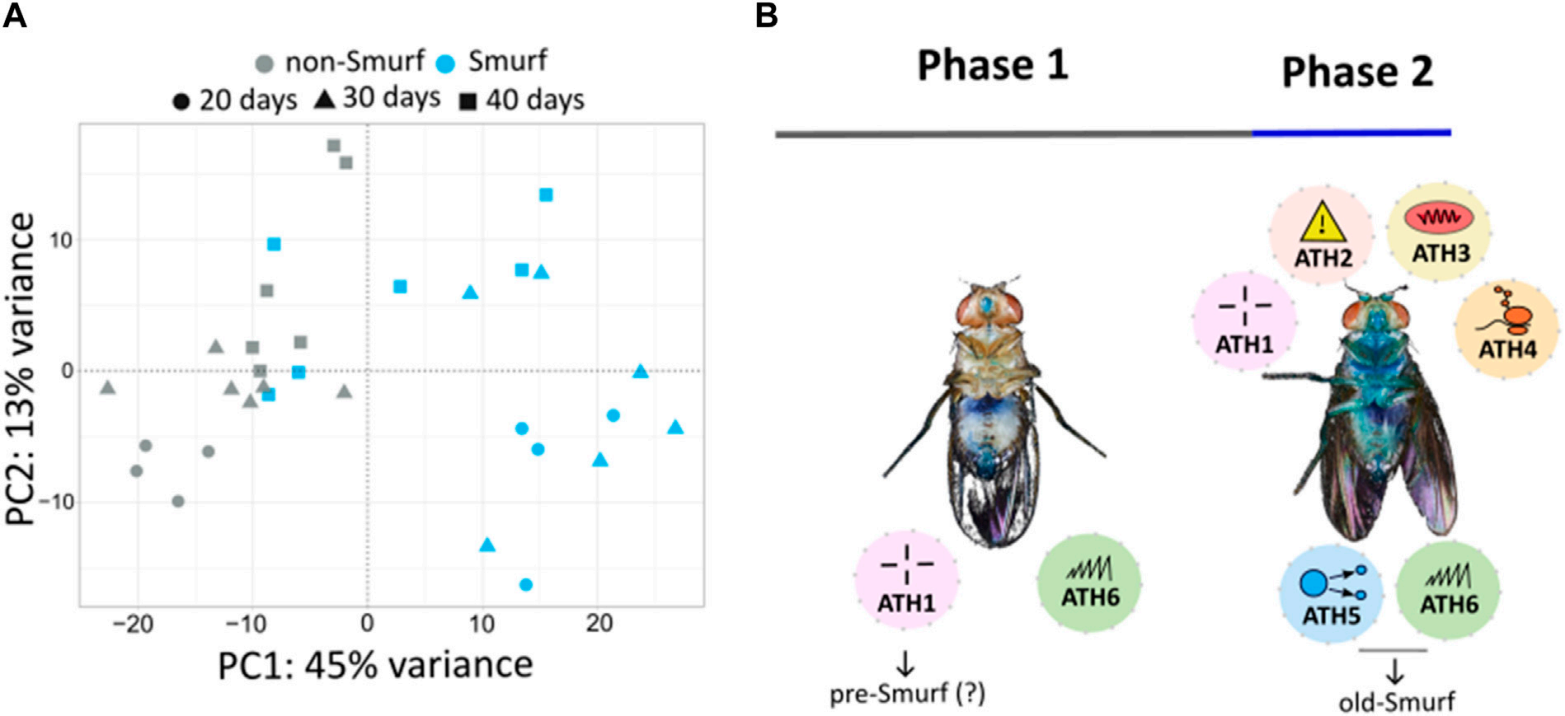
**(A)** Principal Component Analysis (PCA) performed on RNAseq data from whole body Smurfs and non-Smurfs mated females at different ages shows a clear separation of Smurf (blue) and non-Smurf (grey) samples on PC1, while samples are distributed according to age (indicated by shapes as illustrated in the legend) on PC2. Smurfness explains most of the transcriptome variance (45% for PC1), followed by age (13% for PC2). **(B)** Ageing Transcriptional Hallmarks (ATH)—as enunciated by Frenk and Houseley (2018)—show a biphasic distribution with Smurfness. Smurfs carry four out of six markers independently of chronological age, while the remaining two are identified in old Smurfs (10% survival). The only marker displaying progressive increase with chronological age in both non-Smurfs and Smurfs is transcriptional noise (included in ATH6), while non-Smurfs show increased inflammation (ATH1) only at very old age (10% survival), suggesting this could be a pre-Smurf marker. ATH1: dysregulation immune genes; ATH2: stress (and dna damage) response; ATH3: downregulation of mitochondrial proteins; ATH4: ribosomes downregulation; ATH5: reduction of growth factor; ATH6: dysregulation in gene expression.

The metabolomic signature of Smurf and non-Smurf individuals are distinct across five different *Drosophila* lines at their T_50_, when assessed by unsupervised hierarchical clustering (Zane et al., 2023) (Figure 2). In order to identify putative genetic drivers of the metabolic switch, we examined the whole fly transcriptome, as a function of both age and Smurf status, using mated female flies. Smurfs display an age-independent specific signature (Figure 3A) of approximately 3,000 differentially expressed genes (DEGs), representative of 4 (out of 6) transcriptional hallmarks of ageing (Frenk and Houseley, 2018) and not detected in old non-Smurfs (Figure 3B). With this in mind, the Smurf phenotype is a better predictor of an “aged transcriptome” than chronological age itself. The only transcriptional mark detected as progressively altered in non-Smurfs as they age—and conserved in age-matched Smurfs—is the increase in transcriptional noise, computed for each gene as the relative standard deviation across RNAseq biological replicates. We have detected transcriptional inflammation markers in old non-Smurfs (albeit to a much lower extent than Smurfs in comparison to the number of overexpressed genes). These non-Smurfs, however, sampled at 10% survival, have a high risk of turning Smurfs, suggesting that inflammation is either a pre-Smurf marker or an early change that occurs at the transition (Figure 3B).

As time passes for a non-Smurf, its gene expression becomes noisier until it reaches a point—an hypothetical “Smurf Transition Point” yet to be characterised—at which individuals undergo an abrupt modification of the transcriptome, reminiscent of the “transcription hallmarks of ageing” described in Frenk and Houseley (2018) and Zane et al. (2023). This includes the induction of the heat shock response—stress responses in general, previously described in Yang and Tower (2009)—increased inflammation, as well as decreased mitochondrial components—especially the Electron Transfer Chain component—altered control of protein homeostasis, and altered control of gene transcription. These modifications are significant enough to be identified through classic bioinformatics approaches—DESeq2 (Love et al., 2014), GSEA (Subramanian et al., 2005), GO (Ashburner et al., 2000) and KEGG (Kanehisa and Goto, 2000) analyses—in Smurf individuals.

**FIGURE 4:**
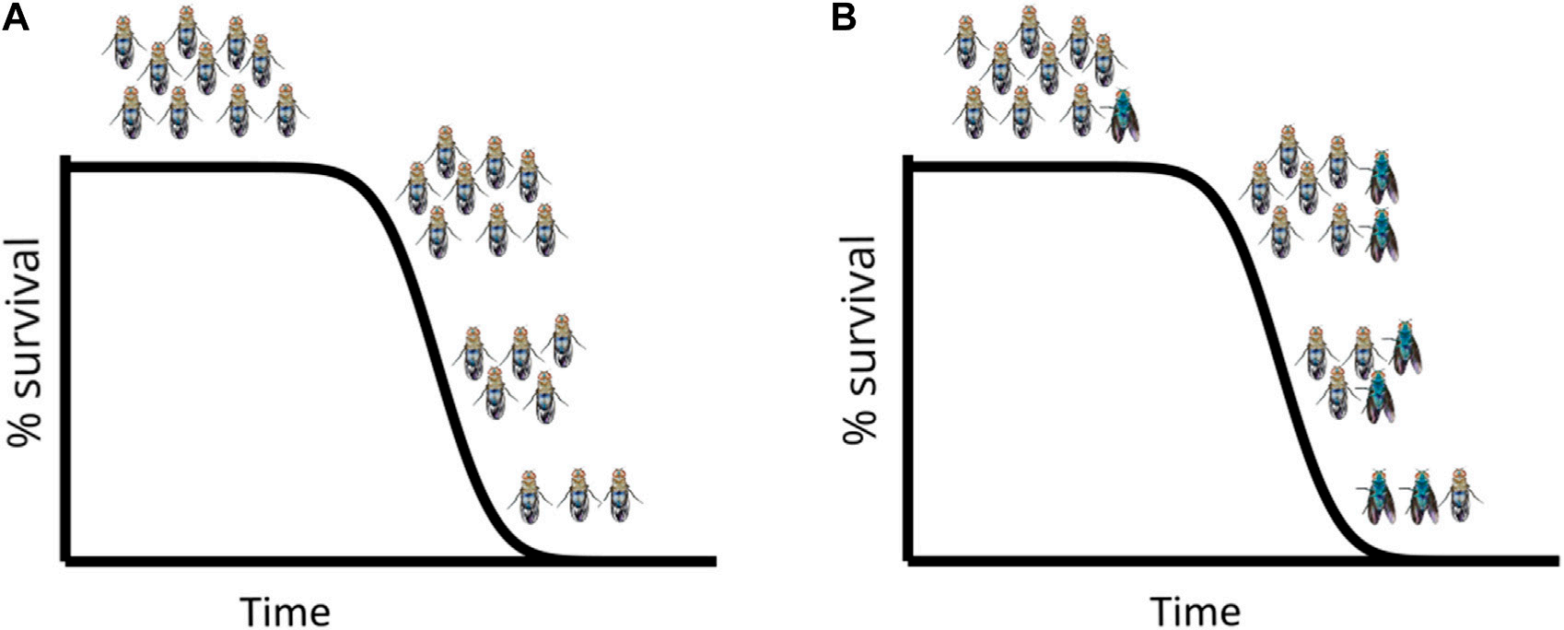
Simulated longevity curves in *D. melanogaster*, on standard food **(A)** and standard food + blue dye FD&C#1 **(B)**: The longevity curve is not altered by the presence of the blue dye which allows identification of Smurf individuals at each time point. The first Smurfs appear in the population towards the end of the survival plateau (i.e., just before the first deaths occur in the population) and from that moment on their proportion (not absolute number) increases with time. Every individual turns Smurf before dying.

It is important to highlight that when sampling individuals at different chronological ages, without performing the Smurf assay—i.e., without discerning Smurfs from non-Smurfs—the broadly used hallmarks of ageing (Lopez-Otin et al., 2013; López-Otín et al., 2023) increase rather progressively with time. However, these hallmarks appear to be abruptly occurring (at the Smurf transition) in a growing proportion of Smurf individuals (Figure 4). At each time point, samples are a mix of s * ***Smurfs_signal*** + (1-s) * ***non-Smurfs_signal*** where *s* is the proportion of Smurfs at the said time. Using the Smurf phenotype to separate chronologically from biologically old individuals amongst a population thus allows us to distinguish the effects of time from that of what we interpret as an end-of-life stereotypical response. This does not exclude the possibility of other changes affecting the hallmarks of ageing as time passes, but they are not statistically detected by conventional approaches. We are able to detect weak changes—using linear modelling of gene expression in non-Smurf samples only—affecting some hallmarks as a function of time (Zane et al., 2023) reminiscent of other early predictors of death recently described in nematodes (Meyer and Schumacher, 2021).

In addition, the deconvolution of this ageing signal allows for the identification of novel regulators of longevity. By targeting putative regulators of candidate transcription factors of Smurf-specific DEGs, we extended—although moderately—the lifespan of treated mated females, delaying the entry into the Smurf phase. It is worth mentioning that the same intervention did not extend lifespan in males, highlighting a sexual dimorphism previously described in ageing *Drosophila* (Malick and Kidwell, 1966; Regan et al., 2016; Belmonte et al., 2020). This does not imply that the biphasic model of ageing is irrelevant to the process of ageing in males. We previously identified Smurfs in males but further studies on the molecular characterization of Smurf males will be needed to clarify this point as well as the sexual dimorphism of this phenotype that was previously described (Rera et al., 2012; Tricoire and Rera, 2015; Dambroise et al., 2016; Martins et al., 2018). Our work suggests that genes involved in the Smurf-specific transcriptional signature represent relevant targets for pro-longevity strategies in a sex-specific manner.

### Using experimentally measured Smurf parameters to model survival curves

**FIGURE 5:**
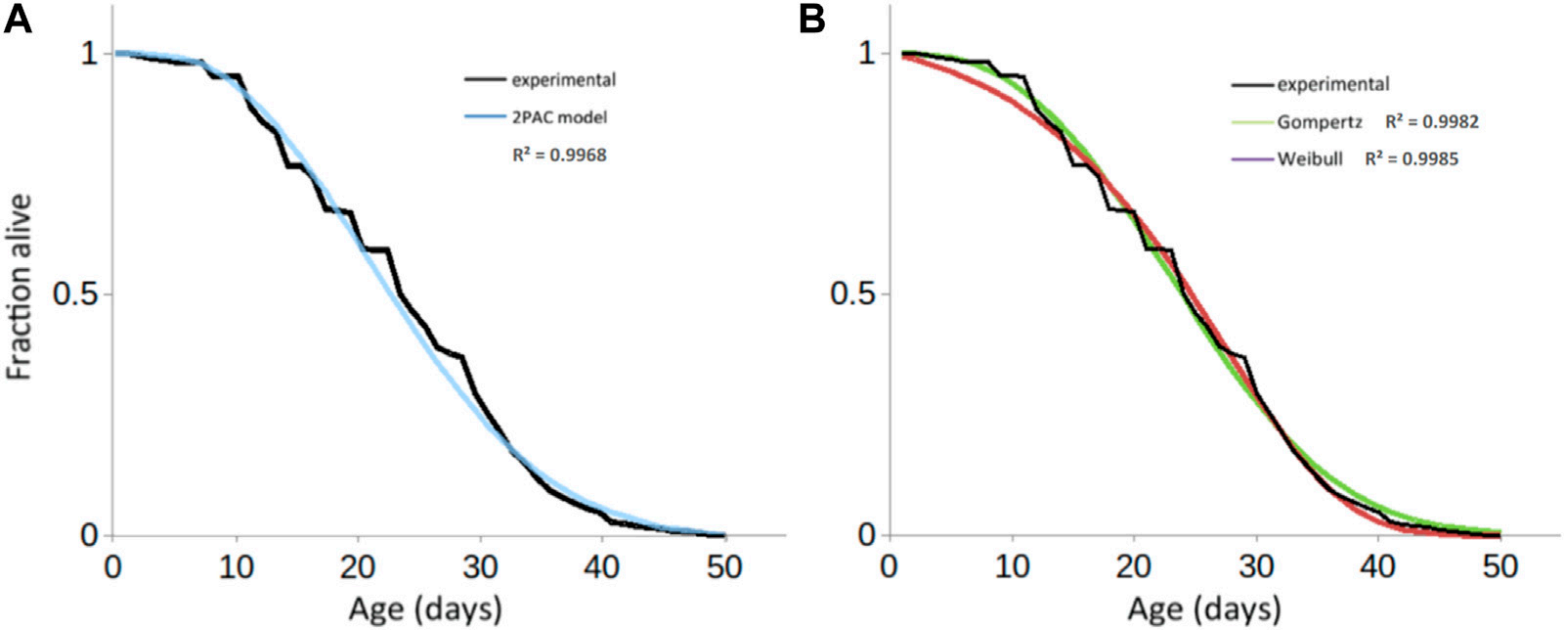
Fitting experimental longevity curve from w^1118^ mated female population using 2PAC, **(A)** Gompertz and Weibull results in similar precision (*a*_2PAC_ = 0.0041; *b*_2PAC_ = −0.012; *R* ^2^ = 0.9968) similar to the fits obtained with either **(B)** the Gompertz model (*a*_Gompertz_ = 0.0061; *k*_Gompertz_ = 0.1029, *R* ^2^ = 0.9982) or the Weibull model (*a*_Weibull_ = 0.000327; *k*_Weibull_ = 2.729554; *R* ^2^ = 0.9985).

The biphasic view of ageing that we are proposing reinterprets the progressive increase in ageing markers as the progressive increase over time of individuals carrying such changes. At each time point in a population, two distinct subpopulations (Smurf and non-Smurf) are present; the resulting longevity curve is made of each subpopulation’s survival trajectory. In 2015, we first proposed a mathematical formulation that stemmed from our experimental observations (2 Phases of Aging mathematiCal model—2PAC model). The model is defined by three, easily biologically-interpretable, as well as experimentally measurable, parameters: ***a*** *,* the rate at which Smurfs appear in a population; ***k***, the rate at which the Smurf population decay; ***t***_***0***_, the time at which the first Smurf appears in the population. Details on the formulation of the model can be found in Tricoire and Rera (2015). Briefly, at any time, the total number of flies in a population is equal to the number of non-Smurfs up to the time ***t***_***0***_, when the first Smurf appears. In flies, this typically occurs a few days before the population exits the survival plateau (i.e., before the first death is observed). From ***t***_***0***_ on, the population experiences a linear increase in the proportion of Smurf at each time point: the speed at which this occurs is defined by parameter ***a***. The Smurf population exponentially decays at a rate ***k***, which is constant regardless of the age of the flies. While ***k*** is also fairly constant across populations (Tricoire and Rera, 2015), ***t***_***0***_ and ***a*** depend on the genetic background of flies. This reflects the recent observation (Zane et al., 2023) that the non-Smurf phase is the plastic phase of ageing—i.e., pro-longevity interventions affect the non-Smurf phase. The apparently strong assumption of linearity for phase 1 and constant duration of the Smurf phase nevertheless allow a reasonable quality of fit compared to classic models of longevity curves (Figure 5B).

**FIGURE 6:**
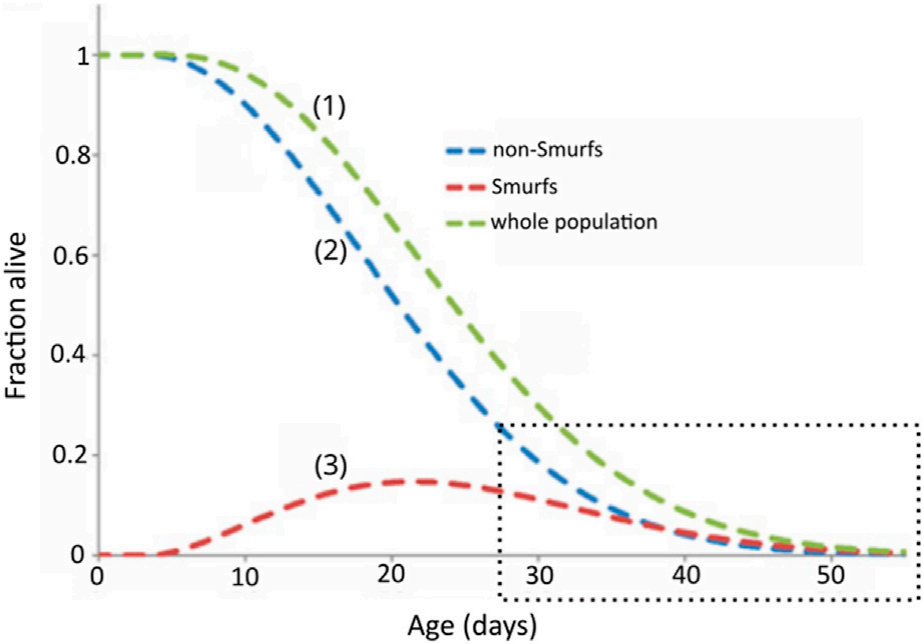
The longevity curve observed for a homogenous population (1) of flies is the sum of two sub-populations, non-Smurfs (2) and Smurfs (3). The behaviour of each curve is defined by the equations presented in Tricoire and Rera (2015). The fraction of alive Smurfs peaks around population mid-life, after which it starts decreasing. The amount of alive Smurfs and non-Smurfs converge at the end of the population lifespan (highlighted by the dotted square). Adapted from Tricoire and Rera (2015).

Classically, the mathematical models used for modelling survival curves and inferring the parameters of the studied population are the parametric Gompertz model—which assumes that the rate of mortality increases exponentially with age—the Weibull model—which assumes that the rate of mortality increases following a power law of age—or the non-parametric Kaplan-Meier and Cox models. The latter focuses on the effect of several explanatory variables (covariates) on a given hazard (or risk of an event, such as death); its key feature is the proportionality of hazards, meaning the effect of the covariates on the risk is multiplicative and does not change over time. One major limitation of these models is the assumption of homogeneity amongst the population, leading to the same distribution of the time-of-event occurrence for each individual. By adding random effects to the time variables, the frailty models take the heterogeneity of natural populations into account (Vaupel et al., 1979) and the debated deceleration in mortality observed in old age is additionally tracked (Barbi et al., 2018; Gavrilov and Gavrilova, 2019). Less prone to low quality data, model organisms such as *D. melanogaster, Ceratitis capitata* (medfly), *Anastrepha ludens* (larger Mexican fruit fly), and *C. elegans* were shown to exhibit a deceleration in mortality at “extreme ages,” i.e., equivalent to after-80 for humans (Vaupel et al., 1998). Our mathematical model directly accounts for this heterogeneity [Figure 6, curve (1)], which is necessary given that the population is a mixture of non-Smurfs [Figure 6, curve (2)] and Smurfs [Figure 6, curve (3)]. While further mathematical characterization is needed**,** it is interesting to notice that the deceleration of mortality in the population occurs when the number of remaining non-Smurfs becomes close to the number of Smurfs, leading to a decrease of longevity curves slope (Figure 6, dotted rectangle). Indeed, the force of mortality at the population level, being that of Smurfs (mostly age-independent) multiplied by the proportion of Smurfs in the population, its upper limit—i.e., when the latter reaches 1—eventually plateaus.

Importantly, the 2PAC implementation confirms the observation-based hypothesis of our two-phase model for ageing (i.e., the mathematical model is compatible with what is observed experimentally). Ageing can be described as a biphasic process affecting all individuals, with an approximately linear age-dependent increasing risk of undergoing the Smurf transition and an age-independent exponential mortality risk. This model prompts interesting questions that serve to deepen our understanding of ageing (e.g., what explains the almost linear time-dependent increasing risk of becoming a Smurf followed by an exponentially increasing risk of dying once Smurf–mathematical properties that seem to be conserved in the mouse (Supplementary Figure S1) (Cansell et al., 2023).

### The evolutionary conservation of the Smurf phenotype prompts us to question ageing’s evolution

Upon characterising the highly stereotyped phenotypes of *Drosophila* Smurfs, we were driven to search for physiologically old individuals, i.e., Smurfs, in natural conditions given the broad evolutionary conservation of the two-phase, Smurf phenotype. Until recent theoretical work (Baudisch, 2005; Williams et al., 2006), “expectations about ageing in wild populations have been influenced by the classic evolutionary theories of ageing and empirical shortcomings” (Roach and Carey, 2014). Therefore, life beyond the controlled setting of the lab is assumedly short, due to predation and hazardous environments, thus casting genes that govern late-life processes as theoretically inconsequential. It is therefore widely accepted that these genes are under weak to no selective pressure. And consequently, ageing exists because of the declining force of selection on late, age-specific traits (Haldane, 1941; Medawar, 1952; Williams, 1957; Hamilton, 1966). A corollary is that ageing is thought to be mostly occurring in protected environments, in which animals survive to ages never seen in the wild.

**FIGURE 7:**
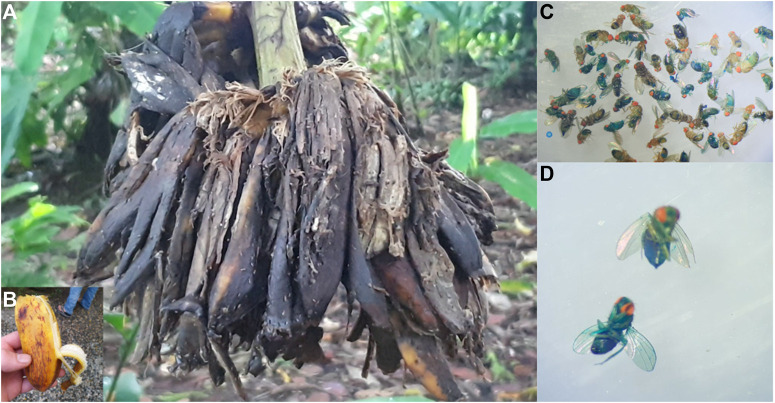
Field work at La Gamba Tropen Station, Costa Rica. **(A,B)** Flies were collected on banana trees in the rainforest. **(C,D)** Wild Smurfs identified through Smurf assay after sampling.

In January 2021, we sampled wild *Drosophila* in the surroundings of the La Gamba Tropen Station, in the Costa Rica rainforest (https://michaelrera.github.io/field_insects_collection/) (Figures 7A, B). Out of a total of 598 flies captured, between 4% and 8% were scored as Smurfs (Figures 7C, D). These results strengthened our belief that the Smurf phenotype is not caused by lab-induced phenomena, and more importantly, that a significant proportion of physiologically old individuals can be found in natural populations. The phenotype of wild Smurf individuals is not yet well-characterised, but this observation immediately raises the question of the role played by old individuals, and their advanced-age gene expression patterns, in natural conditions. More broadly, under what mechanisms has the presence of aged-populations evolved?

**FIGURE 8:**
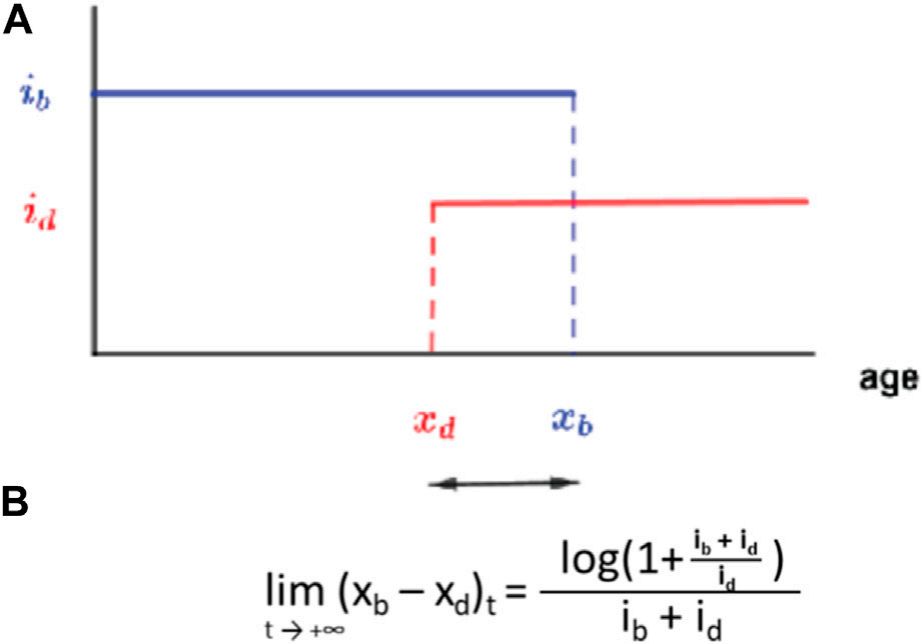
A simple birth-death model for the evolution of ageing as a two-phase process. **(A)** Each individual is defined by an age x_b_ after which fertility is null and x_d_ the age at which the mortality risk becomes non-zero each with a respective intensity parameter. **(B)** Long time limit for the evolution of the trait (x_b_–x_d_). Adapted from Roget et al. (2024).

Ageing—the evolutionary appearance of—is thought to be due to the selection shadow (Haldane, 1941), which, as previously mentioned, allows for the accumulation of late-life mutations (Medawar, 1952) and selection of genes with early benefits associated with late-life negative effects that have not been counter selected (Williams, 1957). In addition, natural populations are not considered to age significantly because of exposure to harsh conditions, [although our field Smurfs (Figure 4) and recent results (Nussey et al., 2013) might challenge this assumption]. Moreover, theoretical analysis recently diminished the putative impact of late-life mutations within the process of ageing (Baudisch, 2005; Dańko et al., 2012). In 2015, we started to develop a novel evolutionary model of ageing that is directly informed by the two-phase ageing model of the Smurf phenotype. This enabled us to both examine the relevance of the two-phase ageing process in evolutionary time and underlying assumptions of the evolution of ageing at large. First, our model is a life-history trait model with overlapping generations in which we define an individual by the core properties of a living organism: 1) its ability to reproduce with variation and 2) its ability to maintain homeostatic integrity (Figure 8A). These two properties are represented respectively by the gene *x*_*b*_—end of fertility—and the gene *x*_*d*_—onset of senescence—in an haploid genome reproducing asexually with independent mutations following a Gaussian distribution centred on the parental gene value.

**FIGURE 9:**
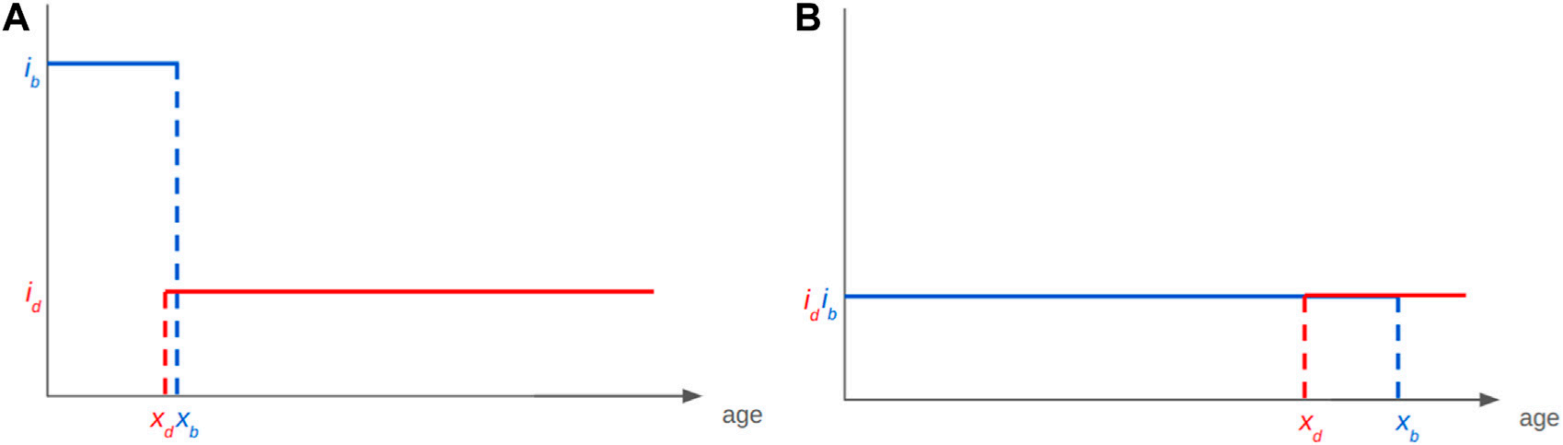
The Smurf-based bd model for the evolution of ageing as a two-phase process explains apparent longevity-fertility tradeoffs. Such trade-off can be explained with the existence of a mathematical constraint on the viability of a given genotype without the need for one on energy allocation. **(A)** A genotype giving high reproductive intensity *i*_*b*_ will require only a short fertility period to get at least one descendant to the next-generation, a small *x*_*b*_. The long time limit of the trait (x_b_–x_d_) being of constant positive value, x_d_ will be small too. **(B)** In the case of a small *i*_*b*_ the opposite will be observed.

Each gene is characterised by a phenotypical intensity, i_b,_ which defines the number of progeny per reproduction event, and i_d_, which defines the probability to die for each death event. We numerically and formally show that such a system will evolve in finite time towards a value of *x*_*d*_ that is slightly inferior to *x*_*b*_ (Roget et al., 2024). In other terms, without imposing any direct constraint between fertility and longevity, these two parameters will converge towards a configuration where the onset of senescence slightly precedes the end of fertility by a duration only defined through the relative intensity of these two characteristics. The significance of this finding is that our model gives way to an elegant explanation for the long-described, although not that widespread (Blacher et al., 2017) longevity-fertility tradeoff supposedly constraining the evolution of ageing. Indeed, the viability of a genotype depends on its ability to give progenies. This creates a mathematical constraint between *i*_*b*_ (the intensity of *x*_*b*_) and *x*_*b*_—much less so between i_d_ and x_d_. The genotype must ensure, at least, the production of one descendant during an individual’s life for its genotype to persist. This means that a genotype with large *i*_*b*_—a high intensity of reproduction, i.e., large number of progeny per unit of time—will require only a short fertility period to ensure its persistence through time. As this minimum condition is fulfilled, *x*_*d*_ is thus constrained to evolve towards *x*_*b*_ by the equation given in Figure 8B. The lifespan of this organism will thus be short (Figure 9A). The opposite will drive the longevity of a genotype with a small *i*_*b*_ (Figure 9B). The model uses binary functions for fertility and senescence but we are studying the impact of more complex functions on the evolution of a given trait, i.e., logarithmic or gamma.

We interpret the end-of-life period between *x*_*d*_ and *x*_*b*_ as reminiscent of the Smurf phase—a late stage of life characterised by high mortality risk and decreased fertility. Strikingly, from the equations of the model, we derive the age-related decrease of selective pressure giving rise to Medawar’s shadow of selection. As such, this model allows us, (by only defining the starting parameters of fertility and homeostatic maintenance of an individual organism and importantly, without imposing any constraint between these two variables) to observe the appearance of thriving individuals albeit (and importantly) with limited life expectancies and limited levels of fertility. Mutations drive the evolution of their lineages towards infinite duration of maintenance and fertility, but only given infinite time. Indeed, because of the decreasing strength of selection, the impact of a given mutation decreases as individuals live longer, making novel mutations less likely to be associated with a significant increase in fitness. The corollary is that, in a finite environment with limited resources, shorter-lived individuals with shorter fertility periods (i.e., individuals that enter the second, “ageing phase” sooner) will show the ability to explore the genotypic landscape faster than a longer-lived line with the same mutation rate (Roget et al., 2024). This is to say that if populations are composed of individuals that age through time, these populations will prove to be more robust because of their ability to explore genotypic space faster. This is what our results suggest, which also allows us to propose that evolution has positively selected for a “function ageing” because of the success it can bring to a population. It is important to note that this modified conception of the evolution of ageing is directly informed by the two-phase ageing process, a pattern that emerged in the model independent of pre-imposed constraints (Méléard et al., 2019).

## Discussion

Conceiving ageing as a continuous, uninterrupted process that unfolds through time and, at large, weakens the physiology of the organism, aligns with the very personal and intimately known experience of human ageing. Despite our experiential perception of ageing, the mechanisms of such a phenomenon are enigmatic; ageing, through an evolutionary perspective, is rather counterintuitive: why and how might a process detrimental to the individual persist through evolutionary time and pervade a wide array of species? Prevailing theories see ageing as somewhat accidental, or a corollary to other things that directly benefit fitness. At the individual level, ageing is largely indistinguishable from the simple passing of time—the organism progressively, and irreversibly declines. Our work suggests otherwise and we aimed to present data that outlines the foundation of a “new ageing framework.”

Through our characterization of the Smurf phenotype (Figure 1) in *D. melanogaster* (Rera et al., 2012), we have demonstrated that the physiological Smurf state encompasses a biological state in which the necessary and sufficient parameters that lead to death are met. Our experiments show that every individual turns Smurf before death, but not all flies do so at the same age, even in isogenic populations. *Drosophila* females turn Smurf on average 3 days before dying (approximately 1 day in males), and physiological reversion to a non-Smurf state has not been observed yet. The risk of becoming a Smurf increases with age, leading to an increase in the proportion (not the absolute number) of Smurf individuals in a population with time (Figure 4). Interestingly, those properties are conserved by Smurfs in *C. elegans* and *D. rerio* (Dambroise et al., 2016) and a short-lived strain of *M. musculus* (Cansell et al., 2023).

All data collected in the past 10 years and presented here support a biphasic model of ageing from flies to mice. According to this framework, an organism ages by following two distinct phases in its life: a first, non-Smurf phase, where the organism is healthy and the risk of mortality is null, yet faces an increasing probability of becoming Smurf with time; and a second, shorter Smurf phase, in which the organism displays the markers of ageing and is committed to death in a specific amount of time (this time depends on the species and is subject to sexual dimorphism).

This framework reinterprets traditional assumptions of ageing (i.e., that the risk of mortality increases exponentially at the population level and that hallmarks of ageing increase progressively through time) and conceptualises the process of ageing as rather something that is constitutive of two phases: a linear one in which the risk of becoming Smurf increases with time and a following exponential phase in which there is an abrupt expression of ageing hallmarks. Our mathematical enunciation of this model removes the assumption of population homogeneity at a given age using parameters that can be quantified experimentally.

Our framework does not invalidate the importance of time in the ageing process. However, it strongly distinguishes between two different “times”: the chronological, calendar one, and the biological one, which is attuned to the individual. Both affect ageing and must be taken into account. Chronological time acts as a risk factor for the entrance into this second phase, while biological time (measured by whether an individual is Smurf or not) more accurately predicts the timing of appearance for the hallmarks of ageing and death. The Smurf model therefore places biological time at the centre of the ageing process, and views chronological time as a covariate.

In our recent study (Zane et al., 2023), we observed that transcriptional noise progressively increases with chronological time in non-Smurfs. This transcriptional noise is not further affected by the biological time of the organism, as Smurfs do not show an increase in transcriptional noise compared to their age-matched counterparts. We therefore hypothesise that an increase in transcriptional noise is one of the mechanisms through which chronological time acts as a risk factor for entrance into the Smurf phase. The non-Smurf phase can thus be conceptualised (within the framework of our model) as a resilience phase, by which the organism can cope with dysfunctions and genetic network alterations up to the moment when perturbations reach a non-tolerance level (Cohen et al., 2022)—i.e., “Smurf Transition Point,” which is yet to be identified—leading to a general stereotyped collapse (i.e., the Smurf phase). A similar view of ageing is supported by the inflammageing theory (Kirkwood and Franceschi, 1992; Franceschi et al., 2000). This theory proposes that ageing is due to “negative hits” affecting the organism’s ageing network, with chronic inflammation being the primary and common hit across individuals. In this view, the causes of collapse can be different in different individuals, but lead to the same outcome (i.e., the onset of the hallmarks of ageing). Recently, it has also been suggested that bioelectric networks act as body coordinators, modulating communications between cells and tissue (Levin, 2023). Ageing would then begin following the miscoordination of such signals, leading to divergence and aberrancies in tissue and organ behaviours (Pio-Lopez and Levin, 2024). Could this be linked to the transcriptional noise increase observed with time? This is, in any case, reminiscent of complex systems theory of ageing and the network theory of ageing proposed 30 years ago (Kowald and Kirkwood, 2016).

The examination of what happens at the moment of the transition, or just before (i.e., the pre-Smurf phase) would help reveal the causes of the organismal collapse. Practically, this is a challenging task, as it requires *in vivo* non-destructive reporters of the individual biological time of pre-Smurfs (i.e., biologically old flies just before they turn Smurf). We hope to identify putative reporter candidates with already available data by further assessing the expression of genes in very old Smurfs (which are statistically enriched in pre-Smurfs) and comparing them to the ones in Smurfs.

Recent work by Bansal et al. (2015) and Zhang et al. (2016) also describes two phases in the context of ageing. However, their conceptualization partially differs from the Smurf model proposed here. Bansal et al. (2015) illustrate two phases based on the organism’s ability to cope with environmental stresses, where interventions increasing lifespan increase the period of sensitivity to those (“gerospan”). Our model, instead, focuses on ageing in physiological conditions; while we can suppose that the “gerospan” phase described is enriched in Smurf individuals, not enough data are available at the moment to affirm that the two are biologically overlapping. In this sense, our work may compare more directly to that of Zhang et al. (2016), as they study physiological ageing in *C. elegans*. It is true that Zhang et al. (2016) describe a rather constant duration of lifespan, while we describe a rather constant duration of time individuals spend in poor health (Smurf phase); however, the scaling they describe seems to be similar to the changes in non-Smurf to Smurf transition rates that we describe between strains with significantly different lifespans (Dambroise et al., 2016). Interestingly, part of their five measured hallmarks might segregate with non-Smurf individuals—i.e., as a function of chronological time only- which would explain why they are able to identify two phases but the last phase is of variable duration.

The Smurf phenotype may not be perfect. As all biological processes, it is a continuous phenotype (Martins et al., 2018), i.e., the release of the blue dye from the intestine is a gradient. Even though there is individual variability in the intensity of the intestinal leakage, we define a Smurf as soon as there is extended coloration visible throughout the body. This binary classification has shown to be relevant both mathematically (Tricoire and Rera, 2015) and molecularly (Rera et al., 2012; Zane et al., 2023). Longevity curves are successfully modelled when it is assumed that the Smurf transition is binary and precedes death within a constant expected remaining amount of time. Finally, individuals who we have been labelled as Smurf show the stereotypical hallmarks of ageing when characterised molecularly. All of this causes reason to believe in the validity of a binary classification.

The assay is simple yet nevertheless presents experimental caveats that may contribute to reproducibility issues. Two teams have reported that only a fraction of individuals turn Smurf prior to death; we feel that addressing this incongruity is best achieved through discussion with these teams, which is an avenue that lies in our future and will undeniably further reveal the unaddressed complexities of ageing (Bitner et al., 2020; Landis et al., 2021; Tower, 2023). It should be recognized, however, that the Smurf assay allows the community of researchers studying ageing to use a physiological, evolutionarily conserved marker, which predicts a high risk of impending death while bringing together other so-called physiological or transcriptional hallmarks of ageing (Rera et al., 2012; Clark et al., 2015; Dambroise et al., 2016; Cansell et al., 2023) even when death is caused by acute stresses (Rera et al., 2012; Katzenberger et al., 2015).

The identification of the two-phase ageing process, something that appears to be highly stereotyped and conserved in *Drosophila*, nematodes, zebrafish and now mice, (reminiscent of what is generally defined as “age-related changes”) prompted us to examine the evolution of ageing. Our resulting evolutionary model shows formally and numerically that an organism capable of reproducing and maintaining itself will necessarily evolve towards a life-history trait demarcated by two consecutive phases: a first healthy phase, followed by a second one of decreased fertility and life expectancy. We interpret the results from this model as reminiscent of the Smurf phase.

In summary, our framework challenges the commonly held belief that ageing markers progressively increase with time in an individual, while reinterpreting this phenomenon as the progressive increase of Smurf individuals within a given population (and as something that has been positively selected for through evolutionary time). Whether the Smurf phase is programmed (and to what extent) remains to be determined. It is nevertheless a highly stereotyped phase both molecularly and physiologically. The evolutionary conservation of ageing, when viewed from the bi-phasal perspective, notably questions the anti-ageing approaches that have driven most of ageing research in the past 20 years. Indeed, if ageing is mostly considered an accident of evolution, why not attempt to “cure” it? If, instead, ageing follows a stereotypical, evolutionarily conserved path, selected for through evolution, can we justify our efforts to seek something that might not, at certain points in time within a lifespan, exist (i.e., a cure)? The framework we propose allows for the identification of factors involved in the risk of entering the second phase of life while providing clues that the latter is likely a phase of chronic disease and higher risk of impending death (and also a time in which interventions might prove ineffective). This framework for studying ageing thus favours the future outlining of a unified model of multiple, age-related chronic conditions that might require distinct interventions depending on whether an organism is in the first or the second phase of its life.

## Data Availability

Publicly available datasets were analyzed in this study. This data can be found here: https://onlinelibrary.wiley.com/doi/full/10.1111/acel.13946.

## References

[B1] Ashburner M. Ball C. A. Blake J. A. Botstein D. Butler H. Cherry J. M. (2000). Gene ontology: tool for the unification of biology. The Gene Ontology Consortium. Nat. Genet. 25, 25–29. 10.1038/75556 10802651 PMC3037419

[B2] Baker G. T. Sprott R. L. (1988). Biomarkers of aging. Exp. Gerontol. 23, 223–239. 10.1016/0531-5565(88)90025-3 3058488

[B3] Bansal A. Zhu L. J. Yen K. Tissenbaum H. A. (2015). Uncoupling lifespan and healthspan in *Caenorhabditis elegans* longevity mutants. Proc. Natl. Acad. Sci. 112, E277–E286. 10.1073/pnas.1412192112 25561524 PMC4311797

[B4] Barbi E. Lagona F. Marsili M. Vaupel J. W. Wachter K. W. (2018). The plateau of human mortality: demography of longevity pioneers. Science 360, 1459–1461. 10.1126/science.aat3119 29954979 PMC6457902

[B5] Baudisch A. (2005). Hamilton’s indicators of the force of selection. Proc. Natl. Acad. Sci. 102, 8263–8268. 10.1073/pnas.0502155102 15919822 PMC1140481

[B6] Baumann C. W. Kwak D. Thompson L. V. (2018). Assessing onset, prevalence and survival in mice using a frailty phenotype. Aging 10, 4042–4053. 10.18632/aging.101692 30562163 PMC6326660

[B7] Belmonte R. L. Corbally M.-K. Duneau D. F. Regan J. C. (2020). Sexual dimorphisms in innate immunity and responses to infection in *Drosophila melanogaster* . Front. Immunol. 10, 3075. 10.3389/fimmu.2019.03075 32076419 PMC7006818

[B8] Bitner K. Shahrestani P. Pardue E. Mueller L. D. (2020). Predicting death by the loss of intestinal function. PLOS ONE 15, e0230970. 10.1371/journal.pone.0230970 32287318 PMC7156097

[B9] Blacher P. Huggins T. J. Bourke A. F. G. (2017). Evolution of ageing, costs of reproduction and the fecundity–longevity trade-off in eusocial insects. Proc. R. Soc. B Biol. Sci. 284, 20170380. 10.1098/rspb.2017.0380 PMC5524490 28701554

[B10] Bocklandt S. Lin W. Sehl M. E. Sánchez F. J. Sinsheimer J. S. Horvath S. (2011). Epigenetic predictor of age. PLoS ONE 6, e14821. 10.1371/journal.pone.0014821 21731603 PMC3120753

[B11] Cansell C. Goepp V. Bain F. Todd N. Douard V. Monnoye M. (2023). Two phases model of ageing in mice: towards a better identification of age-related and late-life metabolic decline [Registered Report Stage 1 Protocol]. Available at: https://hal.science/hal-04234744. 10.6084/m9.figshare.23963208.v1 PMC12542011 41126207

[B12] Clark R. I. Salazar A. Yamada R. Fitz-Gibbon S. Morselli M. Alcaraz J. (2015). Distinct shifts in microbiota composition during Drosophila aging impair intestinal function and drive mortality. Cell Rep. 12, 1656–1667. 10.1016/j.celrep.2015.08.004 26321641 PMC4565751

[B13] Cohen A. A. Ferrucci L. Fülöp T. Gravel D. Hao N. Kriete A. (2022). A complex systems approach to aging biology. Nat. Aging 2, 580–591. 10.1038/s43587-022-00252-6 37117782 PMC12007111

[B14] Cohen A. A. Kennedy B. K. Anglas U. Bronikowski A. M. Deelen J. Dufour F. (2020). Lack of consensus on an aging biology paradigm? A global survey reveals an agreement to disagree, and the need for an interdisciplinary framework. Mech. Ageing Dev. 191, 111316. 10.1016/j.mad.2020.111316 32693105 PMC7603428

[B15] Dambroise E. Monnier L. Ruisheng L. Aguilaniu H. Joly J.-S. Tricoire H. (2016). Two phases of aging separated by the Smurf transition as a public path to death. Sci. Rep. 6, 23523. 10.1038/srep23523 27002861 PMC4802314

[B16] Dańko M. J. Kozłowski J. Vaupel J. W. Baudisch A. (2012). Mutation accumulation may Be a minor force in shaping life history traits. PLoS ONE 7, e34146. 10.1371/journal.pone.0034146 22493680 PMC3320907

[B17] Dent E. Kowal P. Hoogendijk E. O. (2016). Frailty measurement in research and clinical practice: a review. Eur. J. Intern. Med. 31, 3–10. 10.1016/j.ejim.2016.03.007 27039014

[B18] de Vries N. M. Staal J. B. van Ravensberg C. D. Hobbelen J. S. M. Olde Rikkert M. G. M. Nijhuis-van der Sanden M. W. G. (2011). Outcome instruments to measure frailty: a systematic review. Ageing Res. Rev. 10, 104–114. 10.1016/j.arr.2010.09.001 20850567

[B19] Eriksson M. Brown W. T. Gordon L. B. Glynn M. W. Singer J. Scott L. (2003). Recurrent *de novo* point mutations in lamin A cause Hutchinson–Gilford progeria syndrome. Nature 423, 293–298. 10.1038/nature01629 12714972 PMC10540076

[B20] Faya-Robles A. (2018). La personne âgée « fragile. Anthropol. Santé Rev. Int. Francoph. Anthropol. Santé. 10.4000/anthropologiesante.4341

[B21] Franceschi C. Bonafè M. Valensin S. Olivieri F. De Luca M. Ottaviani E. (2000). Inflamm-aging. An evolutionary perspective on immunosenescence. Ann. N. Y. Acad. Sci. 908, 244–254. 10.1111/j.1749-6632.2000.tb06651.x 10911963

[B22] Frenk S. Houseley J. (2018). Gene expression hallmarks of cellular ageing. Biogerontology 19, 547–566. 10.1007/s10522-018-9750-z 29492790 PMC6223719

[B23] Fulop T. Larbi A. Witkowski J. M. McElhaney J. Loeb M. Mitnitski A. (2010). Aging, frailty and age-related diseases. Biogerontology 11, 547–563. 10.1007/s10522-010-9287-2 20559726

[B24] Gavrilov L. A. Gavrilova N. S. (2019). New trend in old-age mortality: gompertzialization of mortality trajectory. Gerontology 65, 451–457. 10.1159/000500141 31295741 PMC6703938

[B25] Gompertz B. (1825). On the nature of the function expressive of the law of human mortality, and on a new mode of determining the value of life contingencies. Phil Trans. Roy. Soc., 513–585. 10.1098/rstb.2014.0379 PMC4360127 25750242

[B26] Haldane (1941). New paths in genetics. Available at: http://archive.org/details/in.ernet.dli.2015.271398 (Accessed October 3, 2019).

[B27] Hamilton W. D. (1966). The moulding of senescence by natural selection. J. Theor. Biol. 12, 12–45. 10.1016/0022-5193(66)90184-6 6015424

[B28] Hannum G. Guinney J. Zhao L. Zhang L. Hughes G. Sadda S. (2013). Genome-wide methylation profiles reveal quantitative views of human aging rates. Mol. Cell 49, 359–367. 10.1016/j.molcel.2012.10.016 23177740 PMC3780611

[B29] Heinze-Milne S. D. Banga S. Howlett S. E. (2019). Frailty assessment in animal models. Gerontology 65, 610–619. 10.1159/000501333 31330523

[B30] Horvath S. (2013). DNA methylation age of human tissues and cell types. Genome Biol. 14, R115. 10.1186/gb-2013-14-10-r115 24138928 PMC4015143

[B31] Horvath S. Raj K. (2018). DNA methylation-based biomarkers and the epigenetic clock theory of ageing. Nat. Rev. Genet. 19, 371–384. 10.1038/s41576-018-0004-3 29643443

[B32] Kanehisa M. Goto S. (2000). KEGG: kyoto encyclopedia of genes and genomes. Nucleic Acids Res. 28, 27–30. 10.1093/nar/28.1.27 10592173 PMC102409

[B33] Katzenberger R. J. Chtarbanova S. Rimkus S. A. Fischer J. A. Kaur G. Seppala J. M. (2015). Death following traumatic brain injury in Drosophila is associated with intestinal barrier dysfunction. Elife 4, e04790. 10.7554/eLife.04790 25742603 PMC4377547

[B34] Kirkwood T. B. Franceschi C. (1992). Is aging as complex as it would appear? New perspectives in aging research. Ann. N. Y. Acad. Sci. 663, 412–417. 10.1111/j.1749-6632.1992.tb38685.x 1482071

[B35] Kiss M. Kiss A. A. Radics M. Popovics N. Hermesz E. Csiszár K. (2016). Drosophila type IV collagen mutation associates with immune system activation and intestinal dysfunction. Matrix Biol. J. Int. Soc. Matrix Biol. 49, 120–131. 10.1016/j.matbio.2015.09.002 26363084

[B36] Kowald A. Kirkwood T. B. L. (2016). Can aging be programmed? A critical literature review. Aging Cell 15, 986–998. 10.1111/acel.12510 27534524 PMC6398523

[B37] Landis G. N. Hilsabeck T. A. U. Bell H. S. Ronnen-Oron T. Wang L. Doherty D. V. (2021). Mifepristone increases life span of virgin female Drosophila on regular and high-fat diet without reducing food intake. Front. Genet. 12, 751647. 10.3389/fgene.2021.751647 34659367 PMC8511958

[B38] Lemoine M. (2021). The evolution of the hallmarks of aging. Front. Genet. 12, 693071. 10.3389/fgene.2021.693071 34512720 PMC8427668

[B39] Levin M. (2023). Bioelectric networks: the cognitive glue enabling evolutionary scaling from physiology to mind. Anim. Cogn. 26, 1865–1891. 10.1007/s10071-023-01780-3 37204591 PMC10770221

[B40] Livingston D. B. H. Patel H. Donini A. MacMillan H. A. (2020). Active transport of brilliant blue FCF across the Drosophila midgut and Malpighian tubule epithelia. Comp. Biochem. Physiol. A. Mol. Integr. Physiol. 239, 110588. 10.1016/j.cbpa.2019.110588 31648063

[B41] Lopez-Otin C. Blasco M. A. Partridge L. Serrano M. Kroemer G. (2013). The hallmarks of aging. Cell 153, 1194–1217. 10.1016/j.cell.2013.05.039 23746838 PMC3836174

[B42] López-Otín C. Blasco M. A. Partridge L. Serrano M. Kroemer G. (2023). Hallmarks of aging: an expanding universe. Cell 153, 1194–1217. 10.1016/j.cell.2013.05.039 36599349

[B43] Love M. I. Huber W. Anders S. (2014). Moderated estimation of fold change and dispersion for RNA-seq data with DESeq2. Genome Biol. 15, 550. 10.1186/s13059-014-0550-8 25516281 PMC4302049

[B44] Malick L. E. Kidwell J. F. (1966). The effect of mating status, sex and genotype on longevity in *Drosophila melanogaster* . Genetics 54, 203–209. 10.1093/genetics/54.1.203 5961480 PMC1211101

[B45] Martins R. McCracken A. Simons M. Henriques C. Rera M. (2018). How to catch a Smurf? – Ageing and beyond *in vivo* assessment of intestinal permeability in multiple model organisms. BIO-Protoc. 7, e2722. 10.21769/BioProtoc.2722 PMC5812435 29457041

[B46] Medawar P. B. (1952). An unsolved problem of biology. Med. J. Aust. 1, 854–855. 10.5694/j.1326-5377.1953.tb84985.x 13062953

[B47] Méléard S. Rera M. Roget T. (2019). A birth–death model of ageing: from individual-based dynamics to evolutive differential inclusions. J. Math. Biol. 79, 901–939. 10.1007/s00285-019-01382-z 31190269

[B48] Meyer D. H. Schumacher B. (2021). BiT age: a transcriptome-based aging clock near the theoretical limit of accuracy. Aging Cell 20, e13320. 10.1111/acel.13320 33656257 PMC7963339

[B49] Nussey D. H. Froy H. Lemaitre J. F. Gaillard J. M. Austad S. N. (2013). Senescence in natural populations of animals: widespread evidence and its implications for bio-gerontology. Ageing Res. Rev. 12, 214–225. 10.1016/j.arr.2012.07.004 22884974 PMC4246505

[B50] Pio-Lopez L. Levin M. (2024). Aging as a morphostasis defect: a developmental bioelectricity perspective. Preprint: 10.31219/osf.io/wkhx4 38636560

[B51] Regan J. C. Khericha M. Dobson A. J. Bolukbasi E. Rattanavirotkul N. Partridge L. (2016). Sex difference in pathology of the ageing gut mediates the greater response of female lifespan to dietary restriction. eLife 5, e10956. 10.7554/eLife.10956 26878754 PMC4805549

[B52] Rera M. Bahadorani S. Cho J. Koehler C. L. Ulgherait M. Hur J. H. (2011). Modulation of longevity and tissue homeostasis by the Drosophila PGC-1 homolog. Cell Metab. 14, 623–634. 10.1016/j.cmet.2011.09.013 22055505 PMC3238792

[B53] Rera M. Clark R. I. Walker D. W. (2012). Intestinal barrier dysfunction links metabolic and inflammatory markers of aging to death in Drosophila. Proc. Natl. Acad. Sci. U A 109, 21528–21533. 10.1073/pnas.1215849110 PMC3535647 23236133

[B54] Rera M. Vallot C. Lefrançois C. (2018). The Smurf transition: new insights on ageing from end-of-life studies in animal models. Curr. Opin. Oncol. 30, 38–44. 10.1097/CCO.0000000000000419 29064844

[B55] Resnik-Docampo M. Koehler C. L. Clark R. I. Schinaman J. M. Sauer V. Wong D. M. (2017). Tricellular junctions regulate intestinal stem cell behaviour to maintain homeostasis. Nat. Cell Biol. 19, 52–59. 10.1038/ncb3454 27992405 PMC6336109

[B56] Roach D. A. Carey J. R. (2014). Population biology of aging in the wild. Annu. Rev. Ecol. Evol. Syst. 45, 421–443. 10.1146/annurev-ecolsys-120213-091730

[B57] Roget T. (2018). Selection-mutation dynamics with age structure: long-time behaviour and application to the evolution of life-history traits. Université Paris-Saclay (ComUE). Available at: http://www.theses.fr/2018SACLX111 (Accessed October 8, 2021).

[B58] Roget T. MacMurray C. Jolivet P. Méléard S. Rera M. (2024). A scenario for an evolutionary selection of ageing. eLife 13. 10.7554/eLife.92914 PMC11530237 39485277

[B59] Schaum N. Lehallier B. Hahn O. Pálovics R. Hosseinzadeh S. Lee S. E. (2020). Ageing hallmarks exhibit organ-specific temporal signatures. Nature 583, 596–602. 10.1038/s41586-020-2499-y 32669715 PMC7757734

[B60] Statzer C. Park J. Y. C. Ewald C. Y. (2023). Extracellular matrix dynamics as an emerging yet understudied hallmark of aging and longevity. Aging Dis. 14, 670–693. 10.14336/AD.2022.1116 37191434 PMC10187690

[B61] Subramanian A. Tamayo P. Mootha V. K. Mukherjee S. Ebert B. L. Gillette M. A. (2005). Gene set enrichment analysis: a knowledge-based approach for interpreting genome-wide expression profiles. Proc. Natl. Acad. Sci. 102, 15545–15550. 10.1073/pnas.0506580102 16199517 PMC1239896

[B62] Tarkhov A. E. Alla R. Ayyadevara S. Pyatnitskiy M. Menshikov L. I. Reis R. J. S. (2019). A universal transcriptomic signature of age reveals the temporal scaling of *Caenorhabditis elegans* aging trajectories. Sci. Rep. 9, 7368–7418. 10.1038/s41598-019-43075-z 31089188 PMC6517414

[B63] Tower J. (2023). Markers and mechanisms of death in Drosophila. Front. Aging 4, 1292040. 10.3389/fragi.2023.1292040 38149028 PMC10749947

[B64] Tricoire H. Rera M. (2015). A new, discontinuous 2 phases of aging model: lessons from *Drosophila melanogaster* . PLoS One 10, e0141920. 10.1371/journal.pone.0141920 26528826 PMC4631373

[B65] Vaupel J. W. Carey J. R. Christensen K. Johnson T. E. Yashin A. I. Holm N. V. (1998). Biodemographic trajectories of longevity. Science 280, 855–860. 10.1126/science.280.5365.855 9599158

[B66] Vaupel J. W. Manton K. G. Stallard E. (1979). The impact of heterogeneity in individual frailty on the dynamics of mortality. Demography 16, 439–454. 10.2307/2061224 510638

[B67] Warner H. R. (2004). The future of aging interventions: current status of efforts to measure and modulate the biological rate of aging. J. Gerontol. Ser. A 59, B692–B696. 10.1093/gerona/59.7.B692 15304533

[B68] Weismann A. (1882). Ueber die Dauer des Lebens; ein Vortrag. Jena: G. Fischer. Available at: https://www.biodiversitylibrary.org/bibliography/21312.

[B69] Whitehead J. C. Hildebrand B. A. Sun M. Rockwood M. R. Rose R. A. Rockwood K. (2014). A clinical frailty index in aging mice: comparisons with frailty index data in humans. J. Gerontol. A. Biol. Sci. Med. Sci. 69, 621–632. 10.1093/gerona/glt136 24051346 PMC4022099

[B70] Williams G. C. (1957). Pleiotropy, natural selection, and the evolution of senescence. Evolution 11, 398–411. 10.2307/2406060

[B71] Williams P. D. Day T. Fletcher Q. Rowe L. (2006). The shaping of senescence in the wild. Trends Ecol. Evol. 21, 458–463. 10.1016/j.tree.2006.05.008 16766081

[B72] Wong R. Piper M. D. W. Wertheim B. Partridge L. (2009). Quantification of food intake in Drosophila. PLOS ONE 4, e6063. 10.1371/journal.pone.0006063 19557170 PMC2698149

[B73] Yang J. Tower J. (2009). Expression of hsp22 and hsp70 transgenes is partially predictive of Drosophila survival under normal and stress conditions. J. Gerontol. A. Biol. Sci. Med. Sci. 64A, 828–838. 10.1093/gerona/glp054 PMC2709545 19420297

[B74] Zane F. Bouzid H. Sosa Marmol S. Brazane M. Besse S. Molina J. L. (2023). Smurfness-based two-phase model of ageing helps deconvolve the ageing transcriptional signature. Aging Cell 22, e13946. 10.1111/acel.13946 37822253 PMC10652310

[B75] Zhang W. B. Sinha D. B. Pittman W. E. Hvatum E. Stroustrup N. Pincus Z. (2016). Extended twilight among isogenic *C. elegans* causes a disproportionate scaling between lifespan and health. Cell Syst. 3, 333–345. 10.1016/j.cels.2016.09.003 27720632 PMC5111811

