# Supplementary material for: Ageing as a two-phase process: theoretical framework
Source: Front Aging. 2024 Apr 8;5:1378351. doi: 10.3389/fragi.2024.1378351 (PMC11034523; doi:10.3389/fragi.2024.1378351)
Supplement: Supplementary file 1 [file Image1.pdf]

Supplementary figures

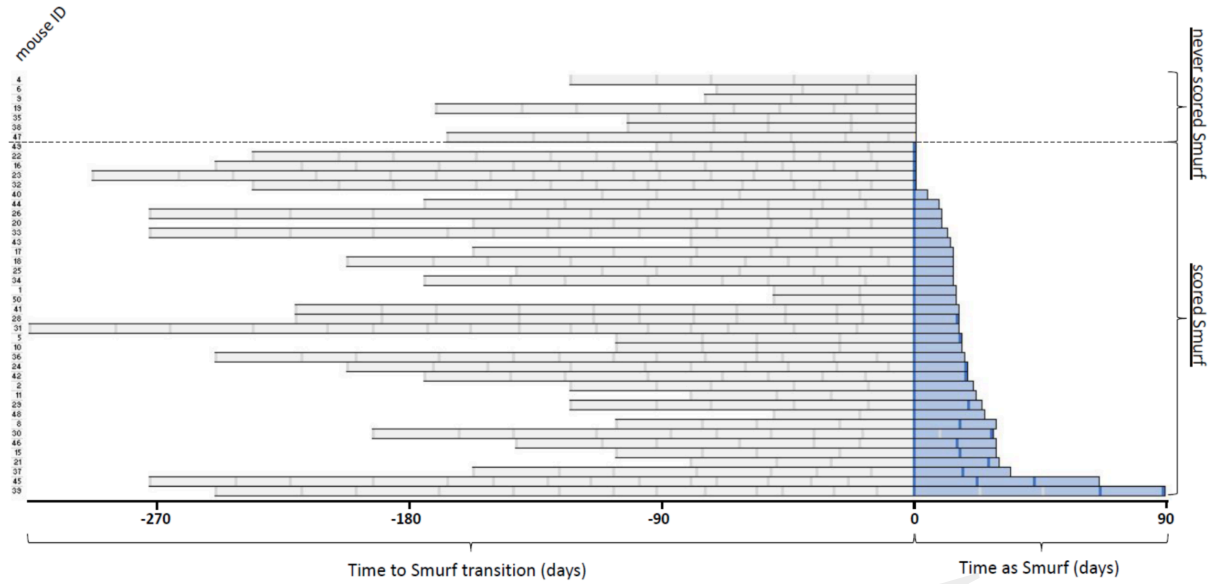

Supplementary figure 1: Time spent as non-Smurf and Smurf in individual AKR/J females followed their whole life. Individuals followed throughout life and assessed for their intestinal permeability as well as multiple physiological parameters were classified *a posteriori* as being non-Smurf (white bars) or Smurf (blue bars) at the end of the experiment. Individuals spend an approximately constant time as Smurfs, much shorter than that as non-Smurfs. Each vertical bar indicates an intestinal permeability measurement. 7 individuals were never scored Smurf, either because they had to be sacrificed for ethics reasons or because they died between two consecutive measurements. *Adapted from* (Cansell et al., 2023).

In review
